# Use and Trends of Diabetes Self-Management Technologies: A Correlation-Based Study

**DOI:** 10.1155/2022/5962001

**Published:** 2022-06-07

**Authors:** Jesús Fontecha, Iván González, Alfonso Barragán, Theodore Lim

**Affiliations:** ^1^MAmI Research Lab. University of Castilla-La Mancha, Ciudad Real, Spain; ^2^School of Engineering & Physical Sciences, Heriot-Watt University, Edinburgh, UK

## Abstract

Applications and systems for diabetes self-management are growing and involve a vast majority of factors to consider. This study was aimed at examining the integration of portable technologies for diabetes self-management, as well as benefits and issues arising of its use. From a web-based study on several groups of people with diabetes, most of them accustomed to the daily use of devices and applications for self-control, a deeper analysis based on correlations and inference was conducted considering information about the disease, technology knowledge and devices handling, use of technologies for diabetes control and management, and training with devices from a clinical and educational viewpoint. In this study, more than 70% of participants use Continuous Glucose Systems and additional devices (41.85% also use insulin pumps) which impacts positively on the knowledge of incoming technologies. The “easy to use” factor of current apps for diabetes self-management is the most valuable feature. Also, 88.98% of participants did not use gamification-based methods during the initial training sessions, although gamification is a useful technique in learning stages. An inference analysis shows how specific characteristics of diabetes devices and apps should improve. On the basis of the results, we discuss about benefits, shortcomings, and the state of these technologies and patient needs for the future.

## 1. Introduction

Advances in mobile computing and embedded sensors demonstrate that systems in the field of chronic and nonchronic diseases are allies in terms of self-management and monitoring. Literature contains a vast majority of studies and works that include technology as the main mechanism of patient control, also with the supervision of clinicians, who are playing an increasingly important role in their use. In diabetes monitoring, the last decade has seen advances in close-loop mobile technology to help patients and clinicians to face the disease.

Glucometers became popular decades ago to measure blood glucose levels instantly, and today, it is still the most widely used form of measurement. However, in recent years, new devices have appeared. Bluetooth-enabled glucometers, Continuous Glucose Monitoring systems (CGM), and other devices are replacing those traditional glucometers with a bunch of new features, such as insulin-dosing advisors and decision support applications [1, 2]. Besides, most of these devices are used along with mobile applications and complete software systems [3]. The global significance of mobile or m-Health applications for remote monitoring and self-management of diabetes is evident in the WHO report [4].

CGM, which has multiple advantages in terms of glucose level monitoring [5], remains a mainstream technology due to showing positive effects and improvements on diabetes self-control as it is presented by Didyuk et al. [1] and Galindo and Aleppo [6] in their evolutionary studies. Although many works are focused on determining the strong and weak points of treatment regimens such as Multiple Daily Injections (MDI) and Continuous Subcutaneous Insulin Infusion (CSII) [7, 8], the use of CGM systems provides benefits in both cases [9]. However, CGM systems also have several barriers such as need for recalibrations, periodic replacement of sensors, rigorous training, and variability in glycemic patterns, mainly related to performance and acceptability [10, 11]. Ideally, CGMs are intended to be used in conjunction with insulin pumps and the appropriate software systems and applications to enhance its capabilities [12, 13]. When integrated, a “closed-loop system” is formed [14]. Besides, there are studies in the literature related to the benefits and shortcomings of other systems like artificial pancreas systems [15], defined by many authors as the “holy grail” of closed-loop systems. Advances in Artificial Intelligence domain provide the basis to achieve an almost complete control on the disease, although the massive use of existing “closed” systems still depends on multiple factors surrounding patients, governments, and their environments [16, 17].

Definitively, taking advantage of these technologies and systems, related aspects to daily life of people with diabetes could also be managed for an optimal control on the disease. It includes remote communication with doctors, scheduling of clinical appointments, improvements in glucose trend-based predictions, or combined control with diet and physical activity [18, 19].

On the other hand, the inclusion of apps in the everyday life of people with diabetes should follow some common standards to ensure a minimum of acceptability and efficiency. However, it is necessary that clinicians are considered to introduce these apps and devices in the clinical practice, during the follow-up of patients, from their diabetic debut as recently proposed by Peters [20]. A good evaluation of usability and user experience standards [21] can help promote the continuous use of mobile applications by users. A qualitative-based approach could lead to an appropriate self-control of diabetes, as well as to the success of these systems [22]. In fact, having focus groups was found to have a significant role in assessing and promoting app acceptance. According to the average age of people with type 1 diabetes, new components such as gamification elements or parent-child tasks have been found to be beneficial [23, 24].

Moreover, clinicians and educators are needed to provide the patient with all the mechanisms and methods for self-management and in using new devices and apps securely [25, 26]. Where the patients are children, parents and family become part of the education procedure [27]. Digitalization and emerging communication mechanisms are changing the diabetes education and the training process. Technological support is essential in each stage of the disease, involving not only the patients but also the family setting. Multimedia content, internet blogs, wikis, or forums are other resources that people with diabetes often use [28], and educators should also take advantage of these in their training sessions.

In this work, we aimed to analyze the knowledge and use of existing technologies and digital support for type 1 and 2 diabetes self-management, which includes smart devices such as glucose meters, CGMs, insulin pumps, or smartphones, as well as software applications focused to monitor blood glucose levels, insulin dosage, diet, and physical activity as main axes involved in diabetes disease from early stages. The results from this analysis are to establish evidence and understand the relationships between usage aspects of software/hardware solutions and its impact on diabetes self-control. This enables us to address the lack of technological knowledge that would pose new challenges for patients, families, health services, and society.

## 2. Materials and Methods

### 2.1. Study Design

Initially, a web-based survey was cocreated in collaboration with clinicians and diabetes specialists and divided into the following five groups of questions:

1. Information about diabetes: it includes general information about the patients and their environment. Also, data about the diabetes debut, type, additional health disorders, and regular control of blood glucose levels are included.
2. Technology knowledge: information about the use of computers and smartphones in the everyday life of patients and the use of health apps focused on sleep quality, diabetes, diet, or physical activity monitoring are included in this block of questions.
3. Diabetes self-management technology knowledge: it focuses on the knowledge and use of technologies for diabetes self-management, for example, the use of blood glucose meters, CGMs, insulin pumps, and artificial pancreas systems and also questions about mobile applications for diabetes control. This is the group with the highest number of questions.
4. Technology in diabetes education and training: in this case, questions about the integration of technology and digital resources during the educational and training process (guided by the educator, as well as by oneself) from the diabetes debut are included.
5. Communication with clinicians: in this block of questions, information regarding the communication mechanisms with the clinicians (primarily endocrines), from diabetes debut to present, was collected.

The groups of questions 3 and 4 involve conditional questions which depend on previous answers from the participants. In this way, only people who use such technologies and/or applications will be asked for more valuable information about these, avoiding the “noise generation” before data preprocessing. The complete template of the questionnaire, with all questions per group, is provided in “Supplementary file 1: Questionnaire Template”.

The web-based survey was open for three months from February to April 2020, with the aim of getting an acceptable sample of participants valid for the consequent data analysis and statement of findings. The web survey link was shared with diabetes communities from different countries.

### 2.2. Recruitment Process

The focus group in this study comprised of people with diabetes (type 1 and 2). During the recruitment process, all the participants were informed about the objective of the study prior to show the survey, and they gave their consent to participate voluntarily through an online form. In case of participants under 16 years old, parents were responsible for accepting the consent form.

Social media groups and associations were the main distribution channels in which the survey was released. This included the following: Facebook private and public groups, Twitter official accounts, and diabetes associations.  Table 1 shows a summary with the number of diabetes communities, associations, and social media groups in which the survey was delivered, according to their main diabetes topics. A detailed list of all the web-based survey distribution channels can be found in “Supplementary file 2: Survey Distribution Channels”.

### 2.3. Data Preprocessing Stages and Preliminary Analysis

Once the web-based survey was closed, demographics from the sample of participants who correctly completed the survey were determined. Then, the focus is placed on studying the significance of several variables from the five groups of questions presented in the Study Design, including both statistical and inferential analyses. Given the heterogeneity of the types of answers to the different questions within the provided groups/blocks, some preprocessing is first required.

Specifically, preprocessing is cleaning up the dataset from the web-based survey to remove all the nonsignificant variables and replacing nonanswered values (NaN) for a numerical default value that can be better handled (0 in this study). This data cleaning and transformation enables preliminary analysis based on the study of correlations on the sample through different methods. Preliminary analysis will reveal if there exist underlying relationships in the sample that can be a suitable starting point for further statistical and inferential analysis (in-depth analysis presented in Results).

Given the variety in the types of answers/responses for each question included in the web-based survey, two correlation approaches have been adopted to deal with.

- Numeric conversion approach: it consists of two steps: (1) transforming all categorical values to quantitative ones, preserving the maximum meaning of the answer; and (2) computing the Spearman rank-order correlation.
- Multicorrelation approach: it consists of directly applying proper correlation methods like Cramer's V [29], point biserial [30], or Spearman's [31] depending on the nature/type of the variable representing each answer.

Processing details of both approaches are included in “Supplementary file 3: Processing Correlation Approaches”.

**Figure 1:**
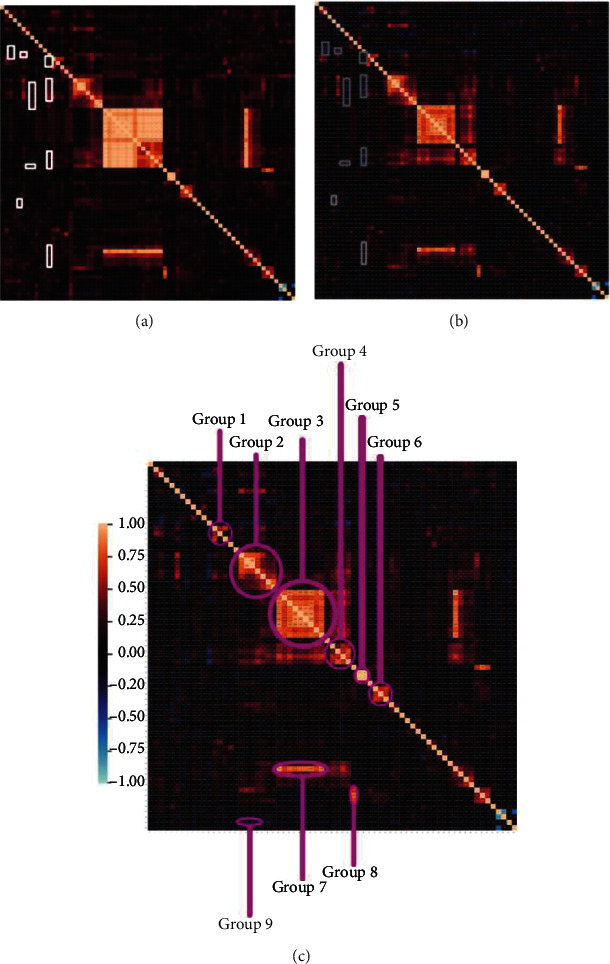
Correlation heat maps and groups. (a) Correlation heat map of multicorrelation analysis. (b) Correlation heat map of numeric conversion. (c) Correlation groups highlighted.

Preliminary analysis on the sample shows similar correlations using the two previous approaches, as illustrated in the general heat maps (including all resulting preprocessed variables) in Figures 1(a) and 1(b). However, numeric conversion approach can retain slightly more meaning from data categorized in intervals due to the preservation of the order relationship presented in the original data. The maintenance of these relationships allows to extract more refining knowledge than using the second correlation approach.

A single proof of the better interpretability and extra information that the data keep within the numerical conversion approach is that it is readily observable medium to low inverse correlations highlighted by grey bounding boxes in Figure 1(b). These correlations highlighted in Figure 1(a) with a white bounding box are weak direct correlations. For instance, an inverse relationship is indicated between the use of Internet search engines to find out information on diabetes and its management (“Did you use Internet Search engines to search information about diabetes and its management?*”*) and the self-consideration of the disease knowledge rated from 1 to 5 (*“*From 1 to 5, How do you rate your knowledge about the disease?”).

The most significant direct and inverse correlations extracted from the responses of the survey's sample are presented in detail in the Statistical Analysis within the Results.

## 3. Results

### 3.1. Demographics and Major Trends in the Use of Technology for Diabetes

**Figure 2:**
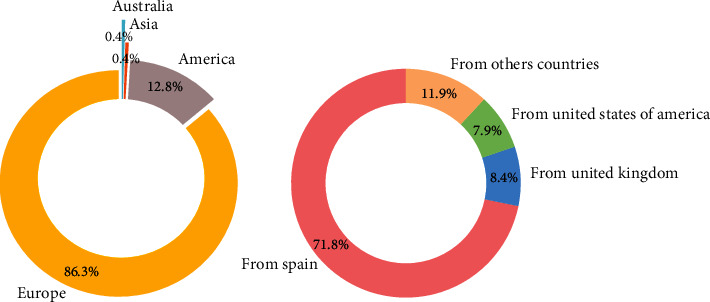
Main percentages of participants by continent and country.

A total of 227 participants completed the survey correctly. Diabetes type 1 was present in 216 participants (95.16%), diabetes type 2 in 8 participants (3.52%), and other types of diabetes accounted for 3 participants (1.32%) of the sample. The average age of the participants was 35, 55 ± 16.62 years. Of these, 30% were male. Since the study started in Spain, most of the participants were people with diabetes from Spain. However, we gathered information from other countries all around the world including people from Australia or Afghanistan. As shown in Figure 2, the most participative countries were Spain (71.8%), UK (8.4%), and USA (7.9%).

In general, people that submitted the survey was mostly female (70%). In terms of age, the sample was slightly more spread than in gender case. More than 50% of the participants were between 30 and 49 years old and less than 2% were over 70.

**Figure 3:**
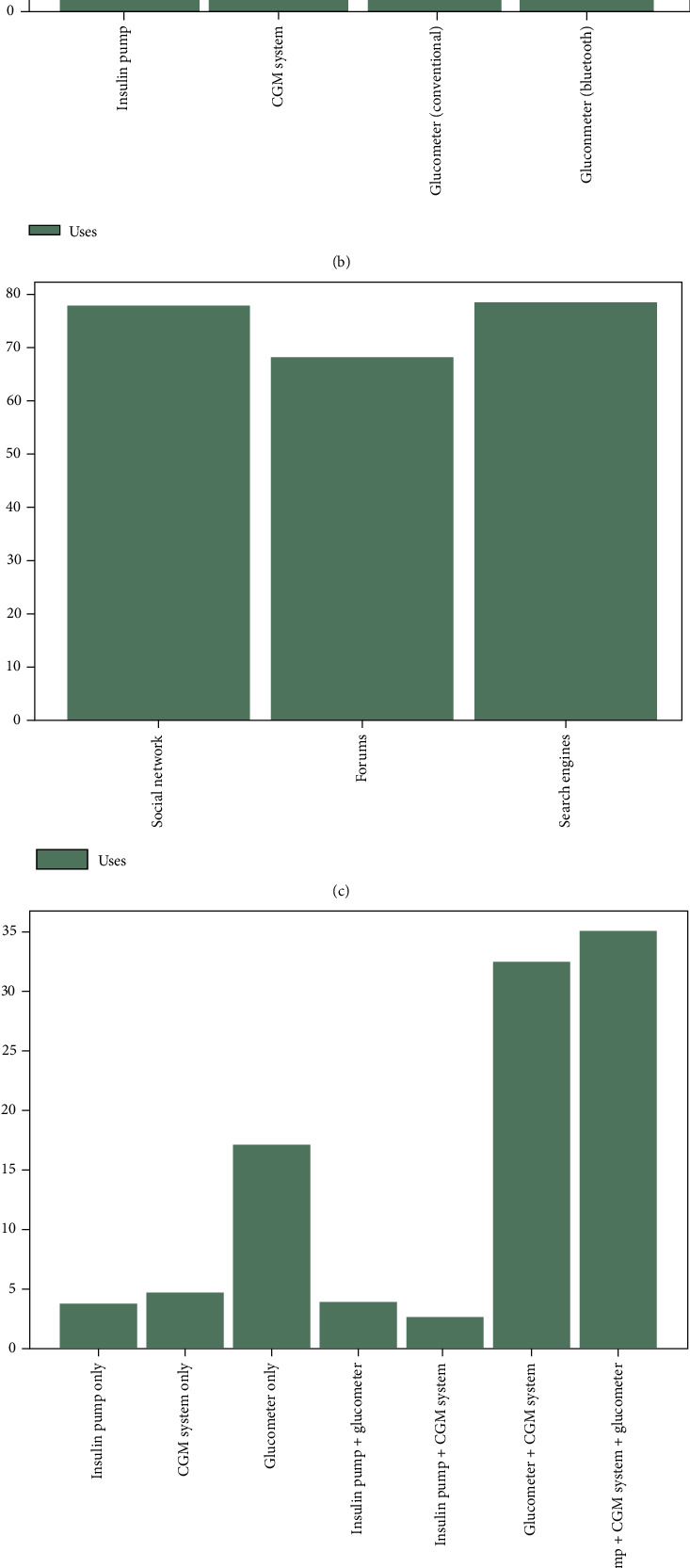
Diabetes management resources. (a) General use of devices. (b) Use of devices for diabetes management. (c) Usage of digital resources for diabetes knowledge. (d) Diabetes devices used during training sessions. (e) Factors considered by apps for diabetes management.

Another important factor shown by this study is the use that participants make of different electronic devices in their daily lives and, specifically, for diabetes management. As can be seen in Figure 3(a), the most used devices were the computer and the smartphone followed by tablets, smartwatches, and fitness wristbands.

Moreover, Figure 3(b) shows the high tendency of using CGM systems in the sample of participants (higher than 70%). Moreover, CGM systems are used in combination with glucometers, 14.9% conventional ones and 17.6% with Bluetooth capabilities. The prevalence of the Bluetooth glucometer over the conventional one in the sample is also an indicator of the importance of the smartphone for diabetes management.

Statistical results from Figures 3(c), 3(d), and 3(e) will be cited in the next section, when the related correlation groups are detailed to understand the context in which they occur.

### 3.2. Statistical Analysis

From the initial correlations found in the sample, and the preliminary analysis, a more in-depth study by using statistical measures was conducted. The focus was on eight visible groups of highly direct correlated values (correlation value greater than 0.5) and another one with the highest inversed correlation that involves technological aspects and/or related to the management of diabetes. Low-strength correlations are not considered because of the possible randomness of the data which makes them very common for the sample size.

From the numeric conversion process and resulting correlation heat maps in Figures 1(a) and 1(b) (see Methods), the groups selected can be observed in Figure 1(c).

**Figure 4:**
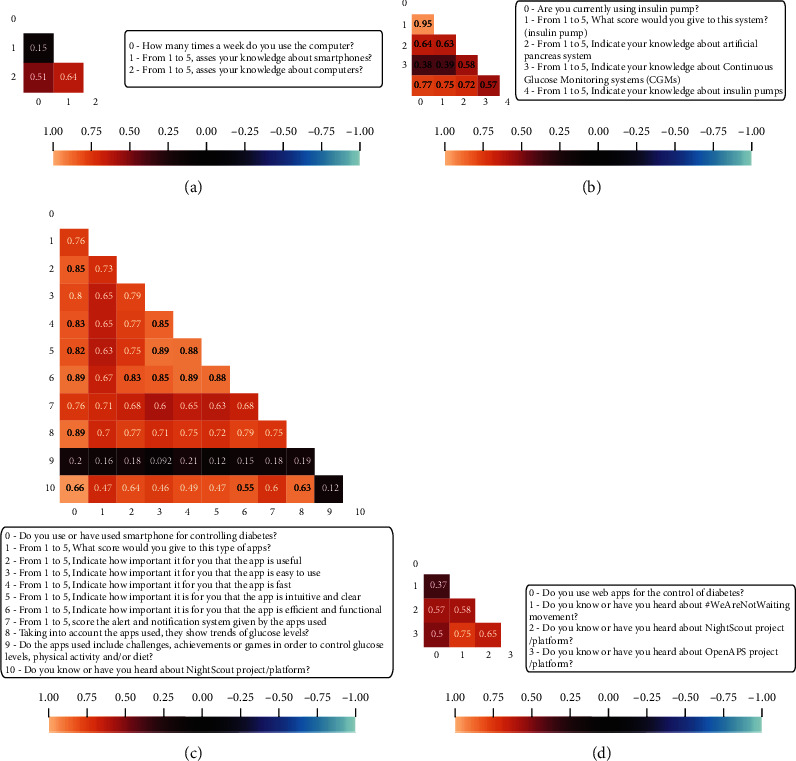
Correlation groups details (first part). (a) G1: digital device correlations; (b) G2: insulin pump correlations; (c) G3: diabetes monitoring through app correlations; and (d) G4: web apps and open projects.

**Figure 5:**
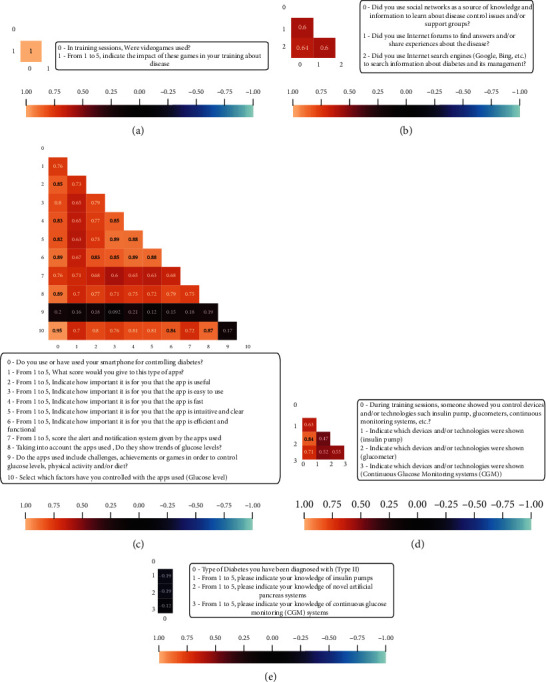
Correlation group details (second part). (a) G5: games during training sessions; (b) G6: diabetes knowledge through digital resources; (c) G7: app characteristics and glucose level monitoring; (d) G8: diabetes devices shown in training sessions; and (e) G9: diabetes type and knowledge on diabetes management devices.

Some of the associations found in the selected correlation groups are based on a specific logical achievement. All correlations group details are presented in Figures 4 and 5.

In G1, shown in detail in Figure 4(a), *frequency of computer use* is related to the *knowledge of the computer* and there is also a direct relationship between increased *knowledge of the computer* and *smartphone expertise.*

G2 group shown in Figure 4(b) gathers a block of correlated questions that deals with insulin pumps, its knowledge, the score given to that system (if used), and certain relationships between knowledge of artificial pancreas and CGMs. About 41.85% of all the survey sample uses an insulin pump, and the average given to this system was 4.27 in a scale from 1 to 5. In fact, the large correlation (0.95) between the use of insulin pump and the high score given to that system is remarkable. The most commonly used was combining insulin pump with CGMs and glucometer with Bluetooth capabilities with a representation of 67.36% from the people that use insulin pumps.

The correlation group G3 shown in Figure 4(c) corresponds to a block of questions related to diabetes monitoring through smartphone apps. The findings suggest a strong relationship between *the desirable characteristics from an app* (intuitive, functional, fast, etc.) and *the score that finally the participants give to the apps that they are currently using*.

**Figure 6:**
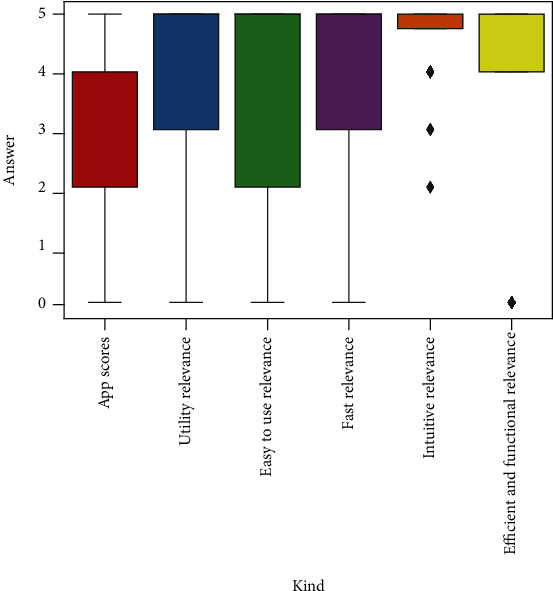
Relevance of desirable usability and user experience characteristics from an app.

Studying these desirable characteristics deeply, “Intuitive*”* and, to a lesser extent, “Efficient and functional*”* are unanimously considered very important by the participants in a desirable app, showing less dispersion as highlighted in Figure 6. The remaining scores are generally distributed between 3 and 5 values.

In G4, a high direct correlation between *using web apps for monitoring diabetes* and the *knowledge about diverse open-source projects* (*OpenAPS*, *NightScout*, etc.) is noticeable (see Figure 4(d)). Perhaps this can be interpreted to mean that participants who typically use web apps and other Information and Communication Technologies (ICTs) for diabetes management and monitoring tasks are more perceptive and more interested in following new projects and initiatives that use technology in the diabetes context.

On the other hand, the G5 group initially included in the statistical analysis was then discarded because of the perfect direct correlation (1.0) between the impact of the application of gamification techniques during diabetic self-management training sessions and their real usage in that phase of learning to live with the disease. The perfect correlation, shown in Figure 5(a), is the result of the presence of only two individuals (a 0.88% of the entire sample) who had used games in their training sessions, and they valued it as very highly in both cases. This unreliable correlation motivated us to look more closely to the use of gamification techniques in diabetes management based on our survey data. We discovered that methods based on gamification to support diabetes management are rarely used, for example, the question “Do the apps used include challenges, achievements or games in order to control glucose levels, physical activity or diet?” had an 88.98% of negative responses.

In G6, relationships between the digital resources used for obtaining information about the disease and its management are presented. These correlations are shown in Figure 5(b). There is a logical association in these results so that participants who are part of social networks related to diabetes management are also likely to be regular participants in forums where they answer questions or share experiences related to the disease and, similarly, they use Internet search engines to look for related information. As illustrated in Figure 3(c), between 68.28% and 78.85% out of the full sample benefit from these kinds of resources, with the use of forums being slightly lower than the other digital resources. Also, looking back at Figure 3(a), it can also be seen that access to these digital resources is mainly done using the smartphone, with a slight advantage over the computer.

G7 group emphasizes the lack of gamification and the use of persuasive techniques to influence glucose levels, physical activity, and/or dietary control in smartphone apps for diabetes management. In response to the question *Do the apps used include challenges, achievements, or games…?* The answers obtained are mostly negative in the survey, as well as uncorrelated with the smartphone use for controlling diabetes, as shown in Figure 5(c) (row corresponding to question number 9). Besides, it also illustrates high direct relations between the use of smartphone apps for controlling diabetes and the desired features to be considered, related to functionality and user experience (*ease of use, speed, intuitive, etc.*). This group also reflects that the main intended use of these apps is to measure glucose levels, among other factors to be controlled.

Regarding this, Figure 3(e) shows a diagram of the different uses of the apps, and the measurement of glucose levels is the most chosen option, with almost twice as many responses as the second most chosen option, which is the administration of insulin. In this regard, the answers given to the question *Select which factors have you controlled with the apps used…* show that participants simply do not rate (a value of 0) the features they do not use.

In the correlation group G8, shown in Figure 5(d), there is an expected correlation that associates the fact of showing one or more diabetes control devices and/or technologies during the training sessions with each of the most common devices that could be presented individually (*insulin pumps*, *glucometers*, and *CGMs*). There were 95 participants who answered “yes” to the question about having been shown devices during the training sessions. From these participants, 76 of their answers pointed to the glucometer, 60 to the CGM and, finally, 51 to the insulin pump. As it is shown, the traditional glucometers were the most presented to the participants, also reinforced by the strong correlation (0.84) with the question about training. Also, a bar chart about the devices used is shown in Figure 3(d), and as pointed out by the correlation group G8, the use of systems together with glucometers and even glucometers alone were the most common combinations showed in training.

In the correlation group G9 (see Figure 5(e)), an inverse relationship can be seen between having diagnosed type 2 diabetes and knowledge about insulin pump devices, CGMs, and artificial pancreas. For future studies, it would be interesting to analyze these questions with a larger sample to corroborate whether there is any causal relationship. At this time, we only have 8 participants with type 2 diabetes so we cannot draw a conclusion about these correlations.

### 3.3. Statistical Inference

In the statistical analysis, high direct correlation rates (> =0.63) were found between the overall rating given to diabetes monitoring and self-management applications used by participants in the sample and the degree of relevance they assigned to certain common usability and user experience features (*usefulness*, *ease*, *quickness*, *efficiency*, etc.). This is mostly presented in the G3 correlation group. Additionally, it was possible to analyze how the usability and user experience features considered were rated by the sample with averages above 3 out of 5 points (see Figure 6). Intuitive, efficient, and functional were the characteristics unanimously considered most important for this type of application.

With the above in mind, we have formulated the following hypothesis for statistical inference:

“Current diabetes monitoring and self-management market apps meets the demands of its users in terms of a compendium of desirable usability and user experience characteristics.”

The first step has been to elaborate a compound index that summarizes all the information. The index has been named *exigence index* as it incorporates, for each participant in the sample, his/her satisfaction score on the applications used regularly in the context of diabetes, as well as the degree of relevance he/she gives to each of the usability and user experience characteristics in these kinds of applications. The exigence index is shown in the Formula 1.

Exigence index:

(1) $$\begin{array}{c} \text{Exigence Index}=\sqrt{{\left(\frac{1}{\text{d}}\overset{\text{d}}{\underset{\text{i}=1}{\sum }}{\text{x}}_{\text{i}}-{\text{Score}}_{\text{conf}}\right)}^{2}} \end{array}$$

where *d* represents the score of the set of characteristics to assess from each participant, and Score_conf_ is the score given to the apps currently using.

The exigence index measures the distance between what the user demands from a useful app for diabetes monitoring and self-management (in terms of usability and user experience) and with what he/she must finally settle for and use, i.e., diabetes applications currently available on the market. To ascertain that distance, it is necessary to compute the difference between the mean of the relevance scores for all the assessed characteristics which represents what the user demands for such kind of apps (first operand) and the score provided to the app/s currently in use (second operand Score_conf_). The resulting difference will be in the range [0, 4], where zero implies that the participants use exactly what they consider essential for an app to manage Diabetes disease (fast, easy to use, etc.) and four means that they use elements far removed from their needs.

Relationships between statistical variables based on the distance measurements are commonly used in a high variety of problems, for example, the *Levenshtein distance* [32] is applied to check the difference between Deutschland's dialects. A more complex compound index based on distance measurement was proposed by Cilla et al. [33] to validate radiotherapy machines.

**Figure 7:**
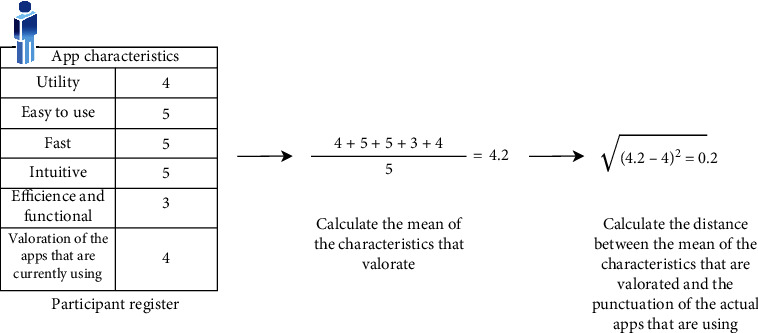
Example of exigence index computation.

Figure 7 illustrates an example of how the compound index is computed step by step. As can be seen, a result of 0.2 indicates that the characteristics scored by the participant (what he/she demands to the app) are close to his/her rating of the app/s being used, this is because the exigence index can take a value from 0 (the participant consumes exactly what is expected) to 4 (the participant consumes the exactly opposite of what is expected).

The exigence index is a quantitative statistical variable constructed from the full experiment sample of the study on which some method of inference can be applied. Then, a hypothesis test has been performed and its results are described below.

The hypothesis formulated is that the distance between the desired characteristics of diabetes apps and what is finally used is very close. Therefore, it is decided to carry out a low tailed *Z* test redefining the hypotheses as follows:

- Null hypothesis (H0): The exigence index is greater than or equal to 1.
- Alternative hypothesis (H1): The exigence index is less than 1.

Table 2 shows the results of this test. The *p* value obtained is below the significance level set (0.05); therefore, the sample evidence suggests the null hypothesis (H0) is rejected in favor of the alternative hypothesis (H1). The inference concludes that the population exigence index is less than 1 so that, in general, there is very small difference on what a diabetic requires from an app, in terms of usability and user experience, and what is finally used based on the diabetes marketplace of applications at the present time. This assumption, coupled with the fact that the mean assessment score for the apps used was 4.05 (over 5) for the sample of participants. This leads us to believe that users are demanding in their expectations of diabetes management applications and that the current market is mature and can meet the demands.

## 4. Discussion

The analysis performed yields interesting results when each main correlation group is compared with the answers given by the sample to the questions that make it up. The G2 correlation group reflects a clear direct relationship (0.95) between the use of insulin pumps and the degree of satisfaction with these systems. In fact, in this context, those participants who use the insulin pump rate it with a high level of satisfaction above 4 out of 5 points. However, participants' responses indicate that only about 42% of them use an insulin pump. It can be seen from the sample that, although continuous insulin infusion systems are highly valued, they do not achieve a significant rate of use currently (below half of the participants). According to the good acceptance of these systems among the participants who use them, it is a pending task to achieve higher usage rates soon.

CGM system has a much more widespread use (over 75% of the sample). In line with the latter, diabetes self-management applications and software are also widely used. The increase in the deployment of these technologies (CGM systems and diabetes self-management apps) is reasonably driven by the widespread daily use of smartphones among the sample (close to 100% as was presented in Figure 3(a)). In fact, G3 correlation group describes a strong relationship between *the desirable characteristics from an app* (in terms of usability) and the final rating that participants give to the applications they currently use. This assertion prompted the hypothesis testing presented in the Results which allowed us to infer that the degree of maturity reached by diabetes self-management applications and software currently on the market meets users' expectations in terms of overall usability and user experience. To proceed with the inferential analysis, it was necessary to build a new statistical variable called *Exigence Index* to estimate the distance between what the user demands from this kind of apps and with what he/she must finally settle for and use. These results, while positive in the context of the integration of apps and their impact on diabetes management, were analyzed from a usability and user experience perspective.

Therefore, the fact that the inference carried out on the degree of maturity reached by diabetes self-management applications and software currently on the market focuses only on a perspective of usability and user experience aspects is seen as a limitation of the study. For example, the inclusion of emerging technologies such as the use of gamification techniques in diabetes management could not be considered a factor in measuring the maturity of diabetes self-management applications. In fact, there was only the presence of two people out of 227 participants who had used games in their diabetes management training sessions, and they rated it as a very high aspect in both cases. This was reflected in the G5 correlation group (Figure 5(a)) which stated an unreliable perfect direct correlation (1.0), as it was a subsample of less than 1% of the study sample. G5 was discarded. It correlated the impact of the application of gamification techniques during diabetic self-management training sessions and their real usage in that learning stage. In this way, we discovered that methods based on gamification to support diabetes management are, to this day, rarely used, for example, the question “Do the apps used include challenges, achievements or games in order to control glucose levels, physical activity or diet?” had an 88.98% of negative responses from the sample.

Considering that approximately 95% of the sample participating in the study suffer from type 1 diabetes, it is relevant that people that submitted the survey was mostly female (~70%) when practically all statistical studies confirm the male predominance of type 1 in all age ranges, with a male-to-female ratio ranging from 1.5 : 1 to 1.8 : 1 for type 1 diabetes [34, 35]. There is no objective reason for this discrepancy in the data, apart from the fact that in the diabetes communities and social media groups consulted (see Table 1) women outnumber men.

Although the number of participants in the study was not large, we have tried to spread the survey over a broad representation of communities and related social media groups, so that the sample is as heterogeneous as was possible through these media. The information collected is intended to be complete and represents the knowledge and particular situation of each participant in the five representative domains, specified in Methods. However, the fact that the main survey distribution channel is mostly digital, as indicated in the “Supplementary file 2: Survey Distribution Channels”; e.g., social media groups in Facebook and Twitter accounts, as opposed to a few traditional diabetes associations and communities, may have had some influence on the profile of the sample, which is usually more accustomed and receptive to the use of technology. Similarly, it should be noted that type 1 diabetes accounts for most of our sample (95.16%). The fact that this type of diabetes occurs more frequently in children or younger adults, who are generally more accustomed and receptive to the use of new technologies, also exerts some influence on the profile of the sample. With all this in mind, and in terms of diabetes self-management technology, these reasons may explain, for instance, why the percentage of CGM use in the sample (~75%) is significantly higher than the current numbers about its use by type 1 diabetic people living in developed countries, who makes up the majority of our sample. For the latter, the use of CGM by type 1 diabetics is currently (till 2019) around ~48%, according to DeSalvo et al. [36].

The pre-processed survey responses have allowed for a comprehensive descriptive and correlational analysis. From an overall perspective, the positive result of the inference on the degree of maturity reached by diabetes self-management applications ensures that current progress in diabetes portable technologies and related applications provides solvent mechanisms for effective, less invasive, and remote monitoring of people with diabetes, especially, if there is a need to cope with pandemic situations (as in the case today with COVID-19).

## 5. Conclusions

The growth in the use of emerging technologies and measurement systems for diabetes self-management is a reality today. However, considering the limitations presented in this work, as well as its results, we can state that the population need to be more involved in the use and consumption of these systems and apps. Effects such as family incomes, engagement concerns, and flaws in diabetes education using technology, are some examples that impact on the efficient growth of technology for diabetes self-control. Thus, although the technological advances are important, these should be aligned with the appropriate knowledge by patients.

## Figures and Tables

**Table 1:** Short summary of diabetes communities and social media groups distributed by their main topics.

Description. Main topic	Channel	Number
Diabetes management with smart devices (CGMs, insulin pumps, or mobile apps)	Facebook groups	10
Type 1 diabetes support	Facebook groups, associations	9
Type 2 diabetes support	Facebook groups	2
Diabetes support and lifestyle (including type 1 and 2)	Facebook groups, Twitter, associations	12

**Table 2:** Hypothesis contrast summary.

Hypothesis contrast	Parameters	Results
H0 : exigence_index > = 1 H1 : exigence_index < 1 Significance level (*α*): 0.05	$\bar{X}=0.634361$ *S* = 0.775772 *n* = 227	*p* value = 0.0125

## Data Availability

The data used to support the findings of this study are available upon request to authors.

## Supplementary Materials

Supplementary Materials Supplementary file 1: “Questionnaire template”. Supplementary file 2: “Survey distribution channels”. Supplementary file 3: “Correlation approaches”.
