# Supplementary material for: Use and Trends of Diabetes Self-Management Technologies: A Correlation-Based Study
Source: J Diabetes Res. 2022 Jun 7;2022:5962001. doi: 10.1155/2022/5962001 (PMC9197631; doi:10.1155/2022/5962001)
Supplement: Supplementary Materials — Supplementary file 1: “Questionnaire template”. Supplementary file 2: “Survey distribution channels”. Supplementary file 3: “Correlation approaches”. [file 5962001.f1.zip › 5962001.f1/Supplementary1_QuestionnaireTemplate.docx]

# Supplementary file. Survey questions and answers details.

**(p):** *It denotes a conditional question to be shown depending on the previous answer (“Yes answers” from the questions colored in red)*

| **Question Group** | **Question** | **Answers** |
| --- | --- | --- |
| General Information | Participation consent | - Yes, I am over 16 years and I accept to participate in this study voluntarily - Yes, but I voluntarily accept to participate as parent of a child with diabetes (under 16 years old) |
|  | Birth date | - Input text answer |
|  | Gender | - Male - Female |
|  | Country of residence | - United Kingdom (UK) - Afghanistan - Åland - Albania - Algeria - American Samoa - Andorra - Angola - Anguilla - Antarctica - Antigua and Barbuda - Argentina - Armenia - Aruba - Ascension Island - Australia - Austria - Azerbaijan - Bahamas - Bahrain - Bangladesh - Barbados - Belarus - Belgium - Belize - Benin - Bermuda - Bhutan - Bolivia - Bonaire, Saint Eustatius and Saba - Bosnia-Herzegovina - Botswana - Brazil - British Indian Ocean Territory - British Virgin Islands - Brunei Darussalam - Bulgaria - Burkina Faso - Burundi - Cambodia - Cameroon - Canada - Cape Verde - Catalonia - Cayman Islands - Central African Republic - Chad - Chile - China (Peoples Republic of) - Christmas Island - Cocos/Keeling Islands - Colombia - Comoros - Congo (Republic of the) - Cook Islands - Costa Rica - Côte d'Ivoire - Croatia - Cuba - Curação - Cyprus - Czech Republic - Democratic Republic of the Congo - Denmark - Djibouti - Dominica - Dominican Republic - East Timor - Ecuador - Egypt - El Salvador - Equatorial Guinea - Eritrea - Estonia - Ethiopia - European Union - Falkland Islands - Faroe Islands - Fiji - Finland - France - French Guiana - French Polynesia - French Southern and Antarctic Lands - Gabon - Gambia (The) - Georgia - Germany - Ghana - Gibraltar - Greece - Greenland - Grenada - Guadeloupe - Guam - Guatemala - Guernsey - Guinea - Guinea-Bissau - Guyana - Haiti - Heard Island and McDonald Islands - Holland - Honduras - Hong Kong - Hungary - Iceland - India - Indonesia - Iran - Iraq - Ireland - Isle of Man - Israel - Italy - Ivory Coast - Jamaica - Japan - Jersey - Jordan - Kazakhstan - Kenya - Kiribati - Korea (North) - Korea (South) - Kuwait - Kyrgyzstan - Laos - Latvia - Lebanon - Lesotho - Liberia - Libya - Liechtenstein - Lithuania - Luxembourg - Macau - Macedonia (Republic of) - Madagascar - Malawi - Malaysia - Maldives - Mali - Malta - Marshall Islands - Martinique - Mauritania - Mauritius - Mayotte - Mexico - Micronesia (Federated States of) - Moldova - Monaco - Mongolia - Montenegro - Montserrat - Morocco - Mozambique - Myanmar - Namibia - Nauru - Nepal - Netherlands (Holland) - Netherlands Antilles (no longer exists) - New Caledonia - New Zealand - Nicaragua - Niger - Nigeria - Niue - Norfolk Island - Northern Ireland - Northern Mariana Islands - Norway - Oman - Pakistan - Palau - Palestinian Territories - Panama - Papua New Guinea - Paraguay - Peru - Philippines - Pitcairn Islands - Poland - Portugal - Puerto Rico - Qatar - Réunion - Romania - Russia - Rwanda - Saint Helena - Saint Kitts and Nevis - Saint Lucia - Saint Maarten - Saint Vincent and the Grenadines - Saint-Pierre and Miquelon - San Marino - São Tomé and Príncipe - Saudi Arabia - Senegal - Serbia - Seychelles - Sierra Leone - Singapore - Slovakia - Slovenia - Solomon Islands - Somalia - South Africa - South Georgia and the South Sandwich Islands - South Sudan - Soviet Union (USSR) - Spain - Sri Lanka - Sudan - Suriname - Svalbard and Jan Mayen Islands - Swaziland - Sweden - Switzerland - Syria - Taiwan - Tajikistan - Tanzania - Thailand - Togo - Tokelau - Tonga - Trinidad and Tobago - Tunisia - Turkey - Turkmenistan - Turks and Caicos Islands - Tuvalu - Uganda - Ukraine - United Arab Emirates - United Kingdom (UK) - United States of America (USA) - Uruguay - US Virgin Islands - Uzbekistan - Vanuatu - Vatican City State - Venezuela - Vietnam - Wallis and Futuna - Western Samoa - Yemen - Yugoslavia - Zambia - Zimbabwe |
|  | Select your education level | - No studies - Primary estudies - Secondary estudies - Higher education |
|  | Approximate annual income level (given in US Dollars) | - Up to 20.000 USD - Between 21.000 and 35.000 USD - More than 36.000 USD |
|  | Number of people with whom you live in your habitual residence | - I live alone - Between 2 and 4 people - More than 4 people |
| General information about your Diabetes | Age at which you were diagnosed with Diabetes | - Between 0 and 11 years old - Between 12 and 17 years old - With more than 17 years old |
|  | Select your type of Diabetes | - Type I - Type II - Gestational - Other (free answer input) |
|  | From 1 to 5, How do you rate your knowledge about the disease? | - 1 = Null - 5 = Excellent |
|  | Do you need help during your glycemic control? | - No - Sometimes - Always |
|  | Indicate the average of daily blood glucose controls (include the calibration controls in case of having a monitoring device) | - Less than 3 - Between 3 and 5 - More than 5 |
|  | During the last year, indicate an approximate number of hypoglycemias suffered (hypoglycemia is understood at levels lower than 3,88mmol / l) | - Less than 4 - Between 4 and 10 - More than 10 - I do not remember |
|  | Where did most of these episodes of hypoglycemia occur? | - At home - Out of home |
|  | During the last year, indicate the approximate number of severe hypoglycemia suffered (meaning severe hypoglycemia at levels below 2,78mmol / l) | - None - Between 1 and 3 - More than 3 - I do not remember |
|  | Approximately, indicate your last known value of glycosylated hemoglobin (HbA1c) | - Less than 6,5% - Between 6,5% and 6,9% - Between 7% and 7,9% - More than 7,9% |
|  | Select which of these habits and/or complications do you currently suffer | - I usually smoke - I am overweight or obese - I do not usually practise physical exercise - I have high blood pressure - I have high cholesterol levels - Other (free answer input) |
| Knowledge and technology use | From 1 to 5, assess your knowledge about computers | - 1 = Null - 5 = Excellent |
|  | How many times a week do you use the computer? | - None - Several times per week - Daily |
|  | What do you use the computer for? | - Work - Internet and social networks - Games |
|  | From 1 to 5, assess your knowledge about Smartphones | - 1 = Null - 5 = Excellent |
|  | How many times a week do you use the Smartphone? | - None - Several times per week - Daily |
|  | Do you have any of these devices? Please select | - Computer - Mobile phone or Smartphone - Tablet - Smartwatch - Fitness wristband - Other (free answer input) |
|  | Do you use any of these apps in a Smartphone, Tablet or Computer? Please, select which ones | - Diet and nutrition monitoring - Physical activity Monitoring - Sleep Monitoring - Diabetes Monitoring - Other (free answer input) |
| Knowledge and use of technologies in diabetes control and management | Type of glucometer that you are currently using | - Conventional - With Bluetooth capabilities |
|  | From 1 to 5, Indicate your knowledge about insulin pumps | - 1 = Null - 5 = Excellent |
|  | Are you currently using insulin pump? | - Yes - No |
|  | (p) From 1 to 5, What score would you give to this system? | - 1 = Null - 5 = Excellent |
|  | From 1 to 5, Indicate your knowledge about artificial pancreas systems | - 1 = Null - 5 = Excellent |
|  | From 1 to 5, Indicate your knowledge about Continuous Glucose Monitoring systems (CGM) | - 1 = Null - 5 = Excellent |
|  | Are you currently using a Continuous Glucose Monitoring system (CGM) | - Yes - No |
|  | (p) Select which of these devices you use or have used | - Dexcom G - Medtronic Minimed - Abbott Freestyle Libre - Other (free answer input) |
|  | (p) From 1 to 5, What score would you give to this system? | - 1 = Null - 5 = Excellent |
|  | Do you use or have used your Smartphone for controlling the Diabetes? | - Yes - No |
|  | (p) Which of the following mobile apps have you used (or use today) to control your Diabetes? | - Glimp - MySugr - Dexcom Mobile App - Librelink - MiniMed Connect - Glooko - Glucosio - Heatlh2Sync - BG Monitor - Glucose Buddy - One Touch Reveal - SocialDiabetes - FEDEDiabetes - One Drop - OnHealth" - Other (free answer input) |
|  | (p) From 1 to 5, What score would you give to this type of apps? | - 1 = Null - 5 = Excellent |
|  | (p) From 1 to 5, Indicate how important it is for you that the app is useful | - 1 = Null - 5 = Excellent |
|  | (p) From 1 to 5, Indicate how important it is for you that the app is easy to use | - 1 = Null - 5 = Excellent |
|  | (p) From 1 to 5, Indicate how important it is for you that the app is fast | - 1 = Null - 5 = Excellent |
|  | (p) From 1 to 5, Indicate how important it is for you that the app is intuitive and clear | - 1 = Null - 5 = Excellent |
|  | (p) From 1 to 5, Indicate how important it is for you that the app is efficient and functional | - 1 = Null - 5 = Excellent |
|  | (p) From 1 to 5, Score the alert and notification system given by the apps used | - 1 = Null - 5 = Excellent |
|  | (p) Taking into account the apps used, do they show trends of glucose levels? | - Yes - No - I do not know |
|  | (p) Select which factors have you controlled with the apps used | - Glucose level - Physical Activity - Diet - Insulin administration - Other (free answer input) |
|  | (p) Do the apps used include challenges, achievements or games in order to control glucose levels, physical activity and/or diet? | - Yes - No - I do not know |
|  | Do you use web apps for the control of Diabetes? | - Yes, web apps based on the previous mobile apps - Yes, others - No |
|  | Do you know or have you heard about #WeAreNotWaiting movement? | - Yes - No |
|  | Do you know or have you heard about NightScout project/platform? | - Yes - Yes, I am also a user - No |
|  | Do you know or have you heard about OpenAPS project/system? | - Yes - Yes, I am also a user - No |
| Evaluate education and training received about Diabetes, especially during the first few months after debut | During the training sessions, someone showed you control devices and/or technologies such as insulin pumps, glucometers, continuous monitoring systems, etc.? | - Yes - No - I do not remember |
|  | (p) Indicate which devices and/or technologies were shown | - Glucometer - Insulin pump - Continuous Glucose Monitoring System (CGM) - Other (free answer input) |
|  | In training sessions, were videogames used? | - Yes, usually - Yes, sometimes - No - I do not remember |
|  | (p) From 1 to 5, indicate the impact of these games in your training about the disease | - 1 = Null - 5 = Excellent |
|  | In training sessions, was audiovisual material used (videos, audios, other multimedia content, etc.) | - Yes, usually - Yes, sometimes - No - I do not remember |
|  | Did you use social networks as a source of knowledge and information to learn about disease control issues and/or support groups? | - Yes - No - I do not remember |
|  | Did you use Internet forums to find answers and/or share experiences about the disease? | - Yes - No - I do not remember |
|  | Did you use Internet search engines (Google, Bing, etc.) to search for information about Diabetes and its management? | - Yes - No - I do not remember |
|  | Have you attended face-to-face courses and/or camps related to Diabetes and its management? | - Yes - No - I do not remember |
|  | Do you or did you belong to diabetes communities and/or associations? | - Yes, currently - Yes, in the past - No |
| Evaluate communication with clinicians during medical checks | Indicate how often do you have medical checks with the endocrinologist or physician | - Less than 2 per year - Between 2 and 4 per year - More than 4 per year |
|  | During the last medical checks, please indicate the methods in which you have provided data about glucose levels to the endocrinologist or physician | - Notebook - Digital format (e.g., in word o pdf) - Mobile application - Spoken (by voice) |
