# Supplementary material for: Use and Trends of Diabetes Self-Management Technologies: A Correlation-Based Study
Source: J Diabetes Res. 2022 Jun 7;2022:5962001. doi: 10.1155/2022/5962001 (PMC9197631; doi:10.1155/2022/5962001)
Supplement: Supplementary Materials — Supplementary file 1: “Questionnaire template”. Supplementary file 2: “Survey distribution channels”. Supplementary file 3: “Correlation approaches”. [file 5962001.f1.zip › 5962001.f1/Supplementary2_DistributionChannels.docx]

# Supplementary file. Details about web-based survey distribution channels and characteristics.

| **Association or group name** | **Language** | **Channel** | **Description. Main topic** |
| --- | --- | --- | --- |
| Dexcom G6 | English | Facebook group | - Diabetes management with Smart devices (CGMs, insulin pumps, or mobile apps) |
| xDrip | English | Facebook group | - Diabetes management with Smart devices (CGMs, insulin pumps, or mobile apps) |
| Nightscout for medtronic | English | Facebook group | - Diabetes management with Smart devices (CGMs, insulin pumps, or mobile apps) |
| Nightscout España | Spanish | Facebook group | - Diabetes management with Smart devices (CGMs, insulin pumps, or mobile apps) |
| Hablamos de bomba de insulina | Spanish | Facebook group | - Type 1 diabetes support |
| El grupo de los piratas con diabetes | Spanish | Facebook group | - Type 1 diabetes support |
| Dexcom G5, G6 y G4 | Spanish | Facebook group | - Diabetes management with Smart devices (CGMs, insulin pumps, or mobile apps) |
| Android APS España | Spanish | Facebook group | - Diabetes management with Smart devices (CGMs, insulin pumps, or mobile apps) |
| Medtronic 640G, 630G, 670G Insulin Pump System support group, ideas & tips | English | Facebook group | - Diabetes management with Smart devices (CGMs, insulin pumps, or mobile apps) |
| Abott Freestyle libre users | English | Facebook group | - Diabetes management with Smart devices (CGMs, insulin pumps, or mobile apps) |
| Diabetes con Freestyle libre | Spanish | Facebook group | - Diabetes management with Smart devices (CGMs, insulin pumps, or mobile apps) |
| Freestyle libre users | English | Facebook group | - Diabetes management with Smart devices (CGMs, insulin pumps, or mobile apps) |
| Diabetes y prediabetes | Spanish | Facebook group | - Diabetes support and lifestyle (including type 1, 2, and others) |
| Vivo con diabetes tipo 2 | Spanish | Facebook group | - Type 2 diabetes support |
| Familias con diabetes tipo 1 | Spanish | Facebook group | - Type 1 diabetes support |
| **Association or group name** | **Language** | **Channel** | **Description. Main topic** |
| Living with diabetes | English | Facebook group | - Diabetes support and lifestyle (including type 1, 2, and others) |
| Diabetes tipo 1 niños de todo el mundo | Spanish | Facebook group | - Type 1 diabetes support |
| Comunidad de diabetes tipo 1 | Spanish | Facebook group | - Type 1 diabetes support |
| Diabetes Mellitus | Spanish | Facebook group | - Diabetes support and lifestyle (including type 1, 2, and others) |
| Diabetes strong community | English | Facebook group | - Diabetes support and lifestyle (including type 1, 2, and others) |
| Diabetes mundial | Spanish | Facebook group | - Diabetes support and lifestyle (including type 1, 2, and others) |
| Type 1 Diabetes | English | Facebook group | - Type 1 diabetes support |
| Diabetes - Positive Attitude towards Diabetes | English | Facebook group | - Diabetes support and lifestyle (including type 1, 2, and others) |
| Diabetes Friends | English | Facebook group | - Diabetes support and lifestyle (including type 1, 2, and others) |
| Diabetes Support Group by Diabee | English | Facebook group | - Diabetes support and lifestyle (including type 1, 2, and others) |
| Type 1 Diabetes in Scotland | English | Facebook group | - Type 1 diabetes support |
| Type 2 Diabetes UK & Ireland | English | Facebook group | - Type 2 diabetes support |
| Type 1 Diaberes UK | English | Facebook group | - Type 1 diabetes support |
| Diabetes UK | English | Twitter account | - Diabetes support and lifestyle (including type 1, 2, and others) |
| Diabetes UK | English | Association | - Diabetes support and lifestyle (including type 1, 2, and others) |
| Sociedad Española de Diabetes | Spanish | Association | - Diabetes support and lifestyle (including type 1, 2, and others) |
| Asociación castellano-manchega Diabetes | Spanish | Association | - Diabetes support and lifestyle (including type 1, 2, and others) |
| Nightscout Foundation | English | Association | - Type 1 diabetes support |
