# Supplementary material for: Use and Trends of Diabetes Self-Management Technologies: A Correlation-Based Study
Source: J Diabetes Res. 2022 Jun 7;2022:5962001. doi: 10.1155/2022/5962001 (PMC9197631; doi:10.1155/2022/5962001)
Supplement: Supplementary Materials — Supplementary file 1: “Questionnaire template”. Supplementary file 2: “Survey distribution channels”. Supplementary file 3: “Correlation approaches”. [file 5962001.f1.zip › 5962001.f1/Supplementary3_CorrelationApproaches.docx]

# Supplementary file. Correlation approaches details

## Numeric conversion approach

Survey questions were classified according to their answer type as illustrated in the taxonomy in the Figure 1a (see below). The type of each question included in the survey can be consulted in a supplementary file. Depending on which kind of answer the question includes, one of the following forms of pre-processing is applied:

- For the *multi-answer type*, every possible answer is modelled by a new dichotomic variable with values 0 or 1 depending on whether the participant selects this answer or not for this specific question.
- For the *single answer yes/no type*, it is modelled with a single dichotomic variable. If the answer is affirmative, the only variable representing the single answer question is set to 1, otherwise 0 is assigned.
- For the *single answer orderable time references*, possible responses are automatically preordered considering time particles found (e.g., *every day*, *never*, *sometimes*, etc.) from lower to higher incidence. Then, sorted responses are mapped to numerical values giving higher values to the responses with particles that represent higher time incidence. The selected choice is represented by its numerical value according to the order previously established.
- For the *single answer orderable numeric references*, if the selected choice is a specific number, it maintains the same value, but if it is an interval of numerical values connected by language particles (e.g., *More than ...*, *Between… and …, etc.*), these particles are automatically segmented for further treatment. Depending on the language particles identified, a numerical value representative of the interval is computed (i.e., *Between 3 and 7*, so “*Between... and ...”* language particle is identified and the middle point between 3 and 7 values is computed, that will be 5 as representative of this interval in this example of response entry). Then, all representatives are ordered and assigned to each interval the position of its representative. A full example can be seen in Figure 1b (see below). For the question on diabetic diagnosis, three orderable response options/entries are provided. The first option represents the interval between 0 and 11 years (using the language particle "*Between... and ...*"), whose middle value is 5.5; the second possible single answer option also uses the same language particle, with 14.5 being its middle point; finally, the third option uses the particle "*more than*", therefore, instead of the middle point its representative is the value indicated (number accompanying the particle) by adding one unit. Finally, representatives of each option are arranged based on a numerical value assigned to each of them.
- For the *single answer unorderable*, there will be pure categorical variables that cannot be ordered, so the only manner to preprocess it is to proceed in a similar manner as for the multi-answer type, creating a new binary variable for each of the response entries that compose the question. Then, the variable representing the selected response will be labelled with 1.


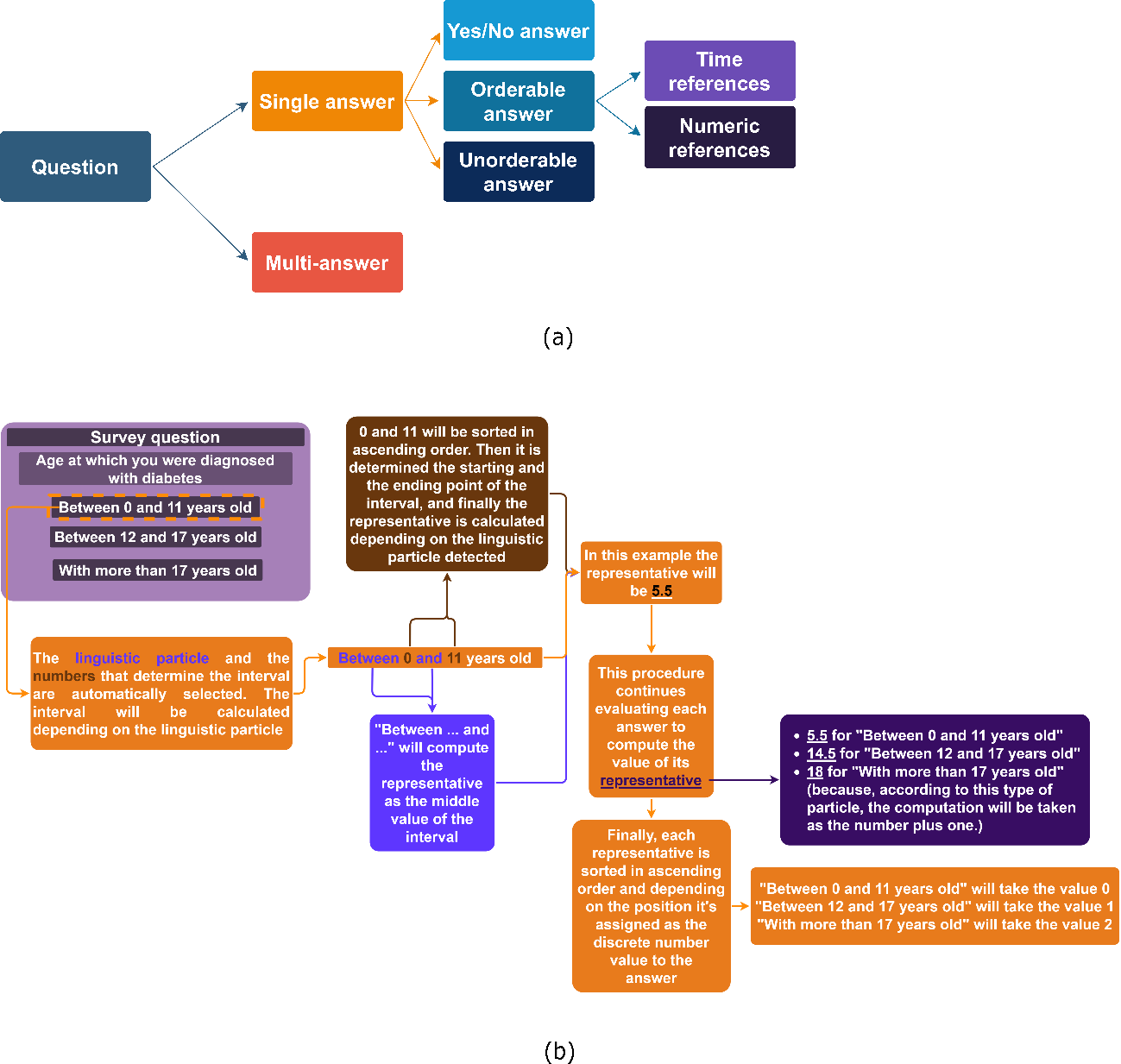


Figure 1. Overview of numeric conversion flow. (a) Answer's taxonomy for numeric conversion method; (b) Processing example: single answer orderable numeric references.

## Multi-correlation approach

In this approach, the answers keep the original type of their variables (categorical or numerical), and then the proper correlation coefficients are computed depending on the nature of the variables involved. The complete process has been summarized in Figure 2 (see below). First, the dataset columns are classified as either categorical or numeric data types. Then, separately, the correlations are computed applying the proper index depending on the nature of each pair of variables/columns (e.g., Cramer's V is used when both variables are categorical/nominal). Finally, all correlation indexes are combined in a single dataset.


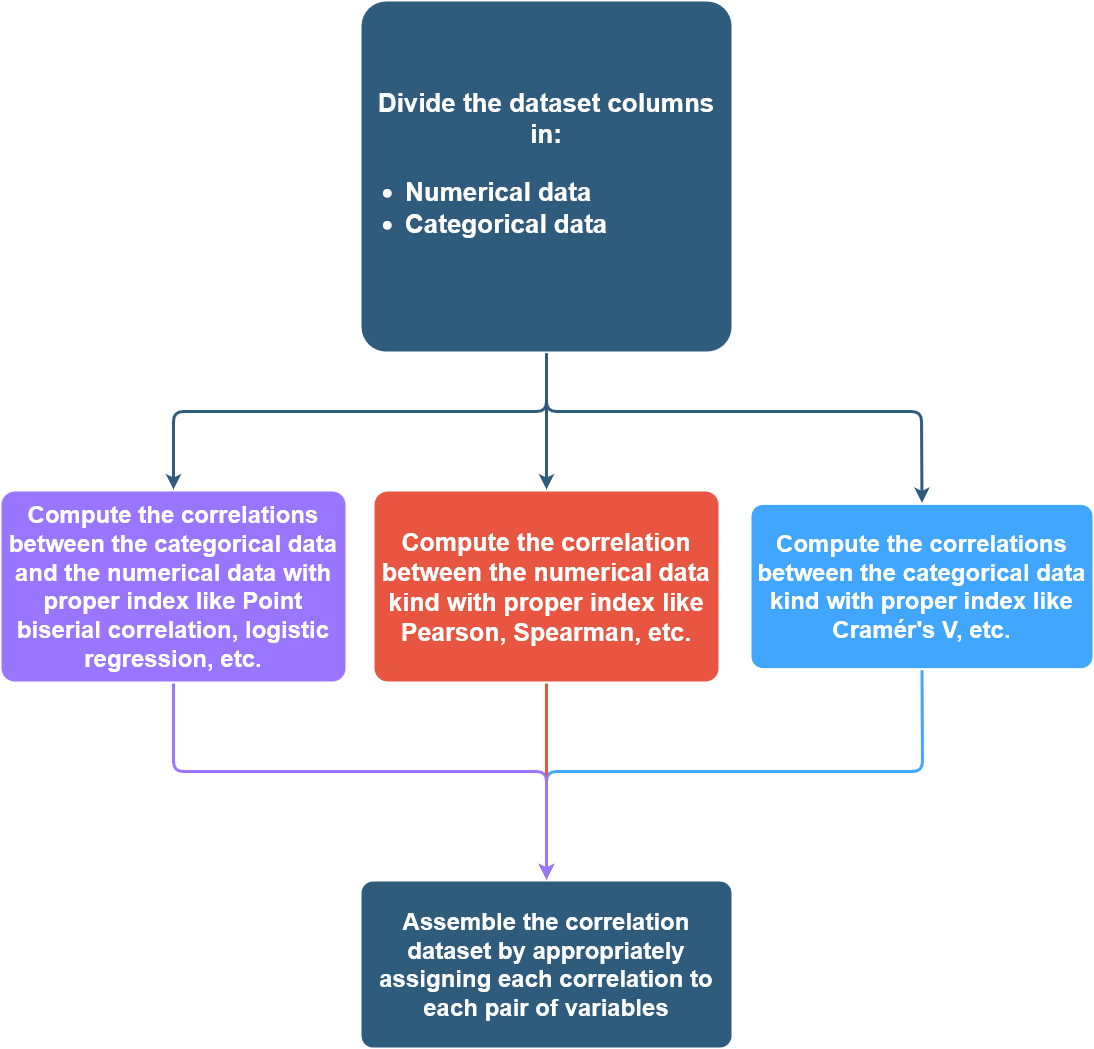


Figure 2. Summary of multi-correlation process
