# Supplementary material for: Use and Trends of Diabetes Self-Management Technologies: A Correlation-Based Study
Source: J Diabetes Res. 2022 Jun 7;2022:5962001. doi: 10.1155/2022/5962001 (PMC9197631; doi:10.1155/2022/5962001)
Supplement: Supplementary Materials — Supplementary file 1: “Questionnaire template”. Supplementary file 2: “Survey distribution channels”. Supplementary file 3: “Correlation approaches”. [file 5962001.f1.zip › 5962001.f1/Supplementary3_Figure2_multicorrelation_approach.pdf]

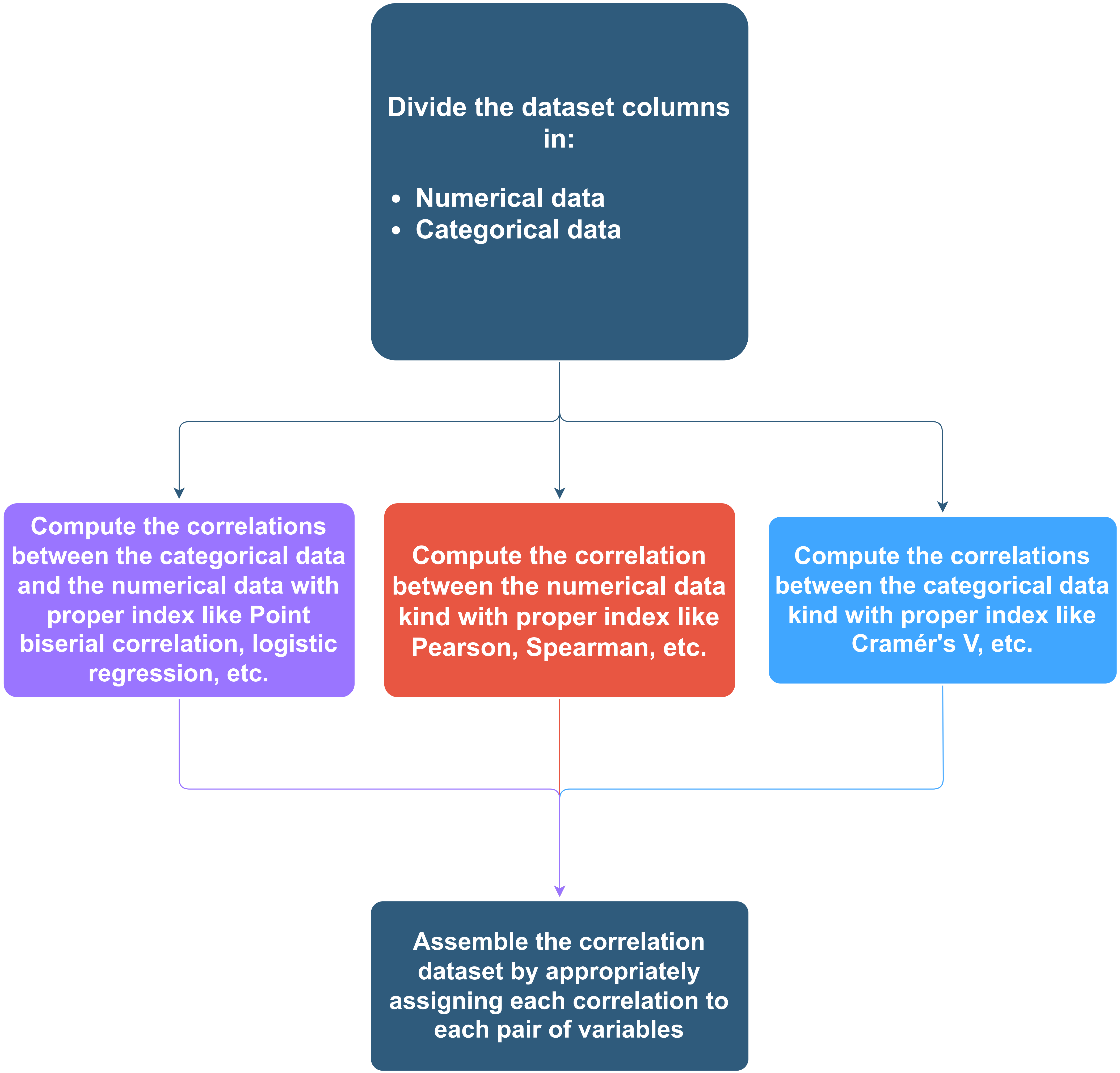
Divide the dataset columns in:
• Numerical data
• Categorical data
Compute the correlations between the categorical data and the numerical data with proper index like Point biserial correlation, logistic regression, etc.
Compute the correlation between the numerical data kind with proper index like Pearson, Spearman, etc.
Compute the correlations between the categorical data kind with proper index like Cramér's V, etc.
Assemble the correlation dataset by appropriately assigning each correlation to each pair of variables
