# Supplementary material for: Use and Trends of Diabetes Self-Management Technologies: A Correlation-Based Study
Source: J Diabetes Res. 2022 Jun 7;2022:5962001. doi: 10.1155/2022/5962001 (PMC9197631; doi:10.1155/2022/5962001)
Supplement: Supplementary Materials — Supplementary file 1: “Questionnaire template”. Supplementary file 2: “Survey distribution channels”. Supplementary file 3: “Correlation approaches”. [file Supplementary3_figure1_numeric_conversion.pdf › 5962001.f1/Supplementary3_figure1_numeric_conversion.pdf]

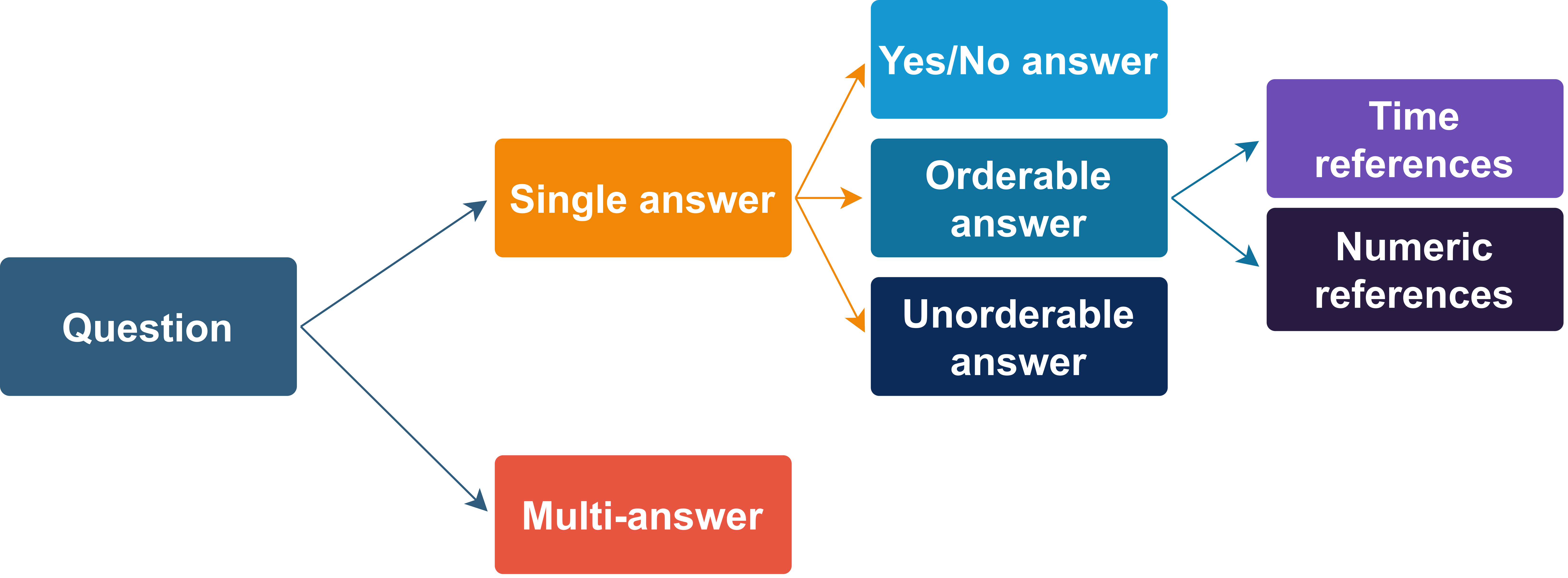

Question
Single answer
Multi-answer
Yes/No answer
Orderable answer
Unorderable answer
Time references
Numeric references

(a)

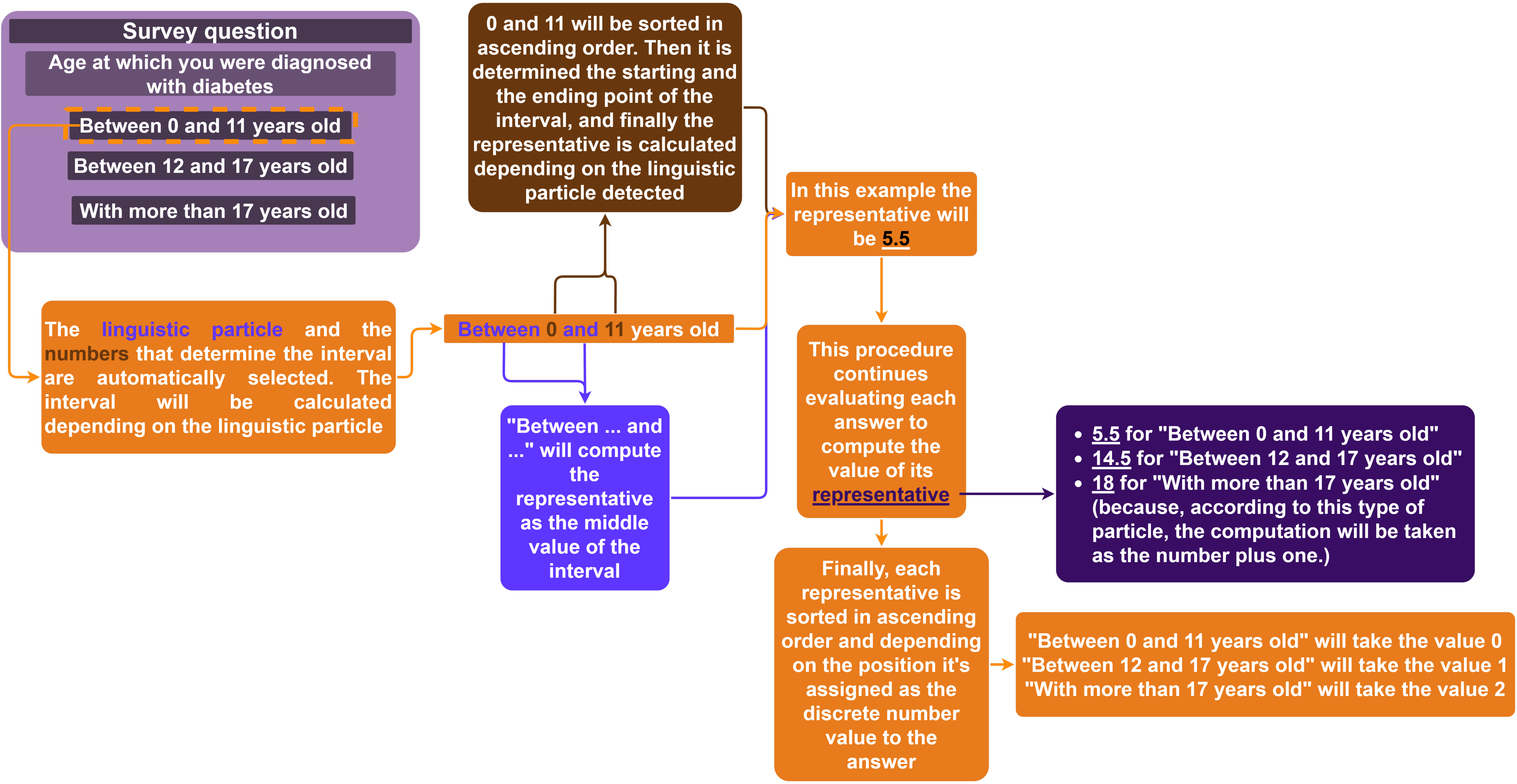

Survey question
Age at which you were diagnosed with diabetes
Between 0 and 11 years old
Between 12 and 17 years old
With more than 17 years old
The linguistic particle and the numbers that determine the interval are automatically selected. The interval will be calculated depending on the linguistic particle
Between 0 and 11 years old
0 and 11 will be sorted in ascending order. Then it is determined the starting and the ending point of the interval, and finally the representative is calculated depending on the linguistic particle detected
"Between ... and ..." will compute the representative as the middle value of the interval
In this example the representative will be 5.5
This procedure continues evaluating each answer to compute the value of its representative
• 5.5 for "Between 0 and 11 years old"
• 14.5 for "Between 12 and 17 years old"
• 18 for "With more than 17 years old" (because, according to this type of particle, the computation will be taken as the number plus one.)
Finally, each representative is sorted in ascending order and depending on the position it's assigned as the discrete number value to the answer
"Between 0 and 11 years old" will take the value 0
"Between 12 and 17 years old" will take the value 1
"With more than 17 years old" will take the value 2

(b)
